# Identifying mutations in Tunisian families with retinal dystrophy

**DOI:** 10.1038/srep37455

**Published:** 2016-11-22

**Authors:** Imen Habibi, Ahmed Chebil, Yosra Falfoul, Nathalie Allaman-Pillet, Fedra Kort, Daniel F. Schorderet, Leila El Matri

**Affiliations:** 1 Institute for Research in Ophthalmology (IRO), Sion, Switzerland; 2 Research Laboratory of Oculogenetic (LR14SP01), Department B of Ophthalmology, Hedi Rais Institute of Ophthalmology, Tunis, Tunisia; 3 Research Laboratory of renal Transplantation and Immunopathology (LR03SP01), University Tunis El Manar, Immunology Laboratory, Tunis, Tunisia; 4 Faculty of Life Sciences, Ecole Polytechnique Fédérale de Lausanne, Lausanne, Switzerland; 5 Faculty of medicine, University Tunis El Manar, Tunisia; 6 Jules-Gonin Eye Hospital, Faculty of biology and medicine, University of Lausanne, Switzerland

## Abstract

Retinal dystrophies (RD) are a rare genetic disorder with high genetic heterogeneity. This study aimed at identifying disease-causing variants in fifteen consanguineous Tunisian families. Full ophthalmic examination was performed. Index patients were subjected to IROme analysis or whole exome sequencing followed by homozygosity mapping. All detected variations were confirmed by direct Sanger sequencing. Mutation analysis in our patients revealed two compound heterozygous mutations p.(R91W);(V172D) in *RPE65,* and five novel homozygous mutations: p.R765C in *CNGB1,* p.H337R in *PDE6B,* splice site variant c.1129-2A > G and c.678_681delGAAG in *FAM161A* and c.1133 + 3_1133 + 6delAAGT in *CERKL*. The latter mutation impacts pre-mRNA splicing of *CERKL*. The other changes detected were six previously reported mutations in *CNGB3* (p.R203*), *ABCA4* (p.W782*), *NR2E3* (p.R311Q), *RPE65* (p.H182Y), *PROM1* (c.1354dupT) and *EYS* (c.5928-2A > G). Segregation analysis in each family showed that all affected individuals were homozygotes and unaffected individuals were either heterozygote carriers or homozygous wild type allele. These results confirm the involvement of a large number of genes in RD in the Tunisian population.

Retinal dystrophies (RD) are a heterogeneous group of diseases in which the photoreceptor and RPE cells of the retina degenerate, leading to partial or complete blindness and affecting approximately 1 in 2500–3500 individuals^1^. Classification of RD is generally based on whether the disease primarily affects cones or rods (predominantly affecting the macula or peripheral retina respectively) and whether it occurs alone (non-syndromic RD) or in conjunction with other systemic disorders, especially loss of hearing (syndromic RD).

RD phenotypes are variable in terms of onset, progression and severity. The disease may be mild and non progressive, such as in congenital stationary night blindness (CSNB), characterized by defective rod photoreceptors involved in night vision. Non progressive disorders may lead to severe visual impairment as well as achromatopsia (ACHM), stationary congenital cone dystrophies. Other disorders are progressive, leading to severe visual impairment such as in retinitis pigmentosa (RP), cone dystrophy (CD), cone-rod dystrophy (CRD)^2^ and Stargardt disease (STGD)^3^. In RP, rod photoreceptors are initially affected more severely than cones^4^. The most severe cases are Leber Congenital Amaurosis (LCA) and early-onset rod–cone dystrophies, in which infants suffer from complete blindness from birth or within the first years of life^5^. Cone dystrophies are characterized by progressive degeneration of cone photoreceptors with preservation of rod function^6^, whereas in CRD peripheral vision is also compromised, leading to early blindness. Stargardt disease is a juvenile macular degeneration characterized by central vision loss^3^.

This group of rare genetic disorders shows substantial clinical and genetic overlaps with high genetic heterogeneity involving more than 220 genes identified so far (https://sph.uth.edu/retnet/). Similar phenotypes may result from different gene mutations, and subtle differences in phenotypes may result from a similar mutation^7^.

The mutations in causative genes induce either in degeneration or dysfunction of retinal cells. Functionally, different gene products are involved in many cellular functions and fall into four categories: proteins directly involved in the phototransduction cascade, genes encoding proteins responsible for the structure and polarity of the photoreceptors, genes encoding proteins of the visual cycle, and regulatory genes (such as transcription and splicing factors)^2^.

All modes of mendelian inheritance have been described in RD, with autosomal recessive being the most prevalent^2^. In our study we focused on the Tunisian population, known to have a relatively high level of consanguineous marriages, leading to a relatively high frequency of autosomal recessive diseases. This study was designed to apply homozygosity mapping in 15 consanguineous Tunisian families segregating retinal degenerative disease and three families analyzed by IROme^7^, aiming at the identification of the genetic defects.

## Materials and Methods

### Subjects

The department B of Hedi Rais Institute of Ophthalmology in Tunisia recruited all subjects involved in the study over a 10-year period. 177 Tunisian families segregating RD were enrolled. In this pilot study, a subset of 61 individuals (32 affected, 29 unaffected) from 15 families with clear recessive transmission and two or more affected individuals was selected. The affected individuals comprised 17 males and 15 females, ranging in age from 4 to 72 years, with an onset of disease ranging from birth to 47 years. Demographic characteristics, age at onset, and personal and family history were recorded for all participants. Written informed consent was obtained from each study participant, analyses were done in accordance with local guidelines and regulations study was approved by the Local Ethics Committee of the Hedi Rais Institute.

All patients underwent a standard ophthalmological examination including determination of best corrected visual acuity using standard Snellen charts. Clinical examination was supplemented by fundus photography, color vision assessment using the Farnsworth-Munsell 100 hue color vision test (FM100, Munsell Color Company Inc., Baltimore, MD, USA) and analysis of dark adaptation. Goldmann kinetic perimetry (Carl Zeiss Meditec Inc., Dublin, CA, USA) using V-4e and I-4e targets, fluorescein angiography (Imagenet; Topcon Corporation, Tokyo, Japan) and optical coherence tomography (SD-OCT) (OCT 3D TOPCON 2000) was also performed. Electrophysiological investigation was carried out using the Métrovision vision monitor (Métrovision, Pérenchies, France) according to the International Society for Clinical Electrophysiology of Vision (ISCEV) protocol.

### Homozygosity mapping and mutation analysis

Genomic DNA of participating individuals was isolated from peripheral blood using a standard salting out procedure. One index patient of 12 families was selected for whole exome sequencing (WES) analysis and unaffected family members were collected for co-segregation analysis. Three index patients were analyzed with IROme^8^.

Exome capturing was performed with the Roche Nimble-Gen version 2 (44.1–megabase pair) at Otogenetics Corporation (Norcross, Georgia) and sequencing was done on an Illumina HiSeq2000 at a mean coverage×31. Sequence reads were aligned to the human genome reference sequence (build hg19), and variants were identified and annotated using the Nextgene software package v.2.3.5. (Softgenetics, State College, PA). Homozygosity was evaluated from SNPs obtained by WES. In families with CERKL mutations, SNPs: rs4667591, rs1050354, rs4894140, rs1143676, rs3754929, rs1400130, rs12476147, rs17228441, rs1800255, rs1225090 were selected and analyzed by Sanger sequencing in the index patients of families F12-F15.

### Assessment of the Pathogenicity of Candidate Variants

We applied various bioinformatic tools to filter variants against NCBI dbSNP to facilitate the identification of disease-causing mutations. The Exome Aggregation Consortium (ExAC; http://exac.broadinstitute.org) data set was used as a reference data set of population-based allele frequencies^9^. Novel DNA variations were compared with data from public databases (HGMD^10^ and 1000 genomes^11^). Although most causative mutations associated with RD are rare (MAF *<* 0.01). The putative pathogenicity of the novel missense variants reported in this study was evaluated using two in silico tools: Polymorphism Phenotyping v2 (PolyPhen-2)^12^ and Sorting Intolerant from Tolerant (SIFT)^13^. The PolyPhen-2 score ranges from 0.0 (tolerated) to 1.0 (deleterious). Variants with scores of 0.0 are predicted to be benign. Values of 1.0 or close to 1.0 are more confidently predicted to be deleterious. PolyPhen-2 and SIFT scores use the same range, 0.0 to 1.0, but with opposite meanings. A variant with a SIFT score of 1.0 is predicted to be benign. In addition, we determined with PolyPhen-2 a measure of evolutionary nucleotide conservation.

### Mutation analysis

After all filtering steps, Sanger sequencing was used to verify each predicted disease-causing variants remaining in the autozygous regions of interest and to perform cosegregation analyses. PCR was realized in a total reaction mixture of 20 μl, containing 20 ng of genomic DNA, 10 pmol of each primer (Eurogentec, Liège, Belgium) and 10 μl of FastStart PCR Master Mix (Roche, Basel, Switzerland). Three amplification steps (30 s each), with the annealing temperature ranging from 58 °C to 60 °C, were performed. Primer sequences were designed using the PRIMER3 software (http://frodo.wi.mit.edu/) and PCR conditions are provided in Table 1. The purified PCR products were then directly sequenced on an ABI 3100XL DNA automated sequencer (Applied Biosystems, Foster city, CA), using the Big Dye Terminator Labeling Kit version 1 (Life Technologies, Zug, Switzerland). Cosegregation analysis was performed in all families.

### Splicing variant analysis

A minigene analysis was done to predict the putative impact of the deletion c.1133 + 3_1133 + 6delAAGT occurring in a splice site. The mutated region was amplified by PCR from DNA of patient VI-1, VI-2 in F13 and one healthy control using specific (forward: 5′-TCC AAA GGA TCC GGG AAC TGA AGA GAA AGT GAG G-3′ and reverse: 5′-TCC AAA GAA TTC GGC TGA GTG AGT AGT TGT TTG C-3′). The resulting PCR products were subsequently cloned into the pBK-CMV vector using the T4 DNA Ligase according to manufacturer’s protocol (Rapid DNA Ligation Kit). Plasmids were analyzed by direct Sanger sequencing and then transfected into HEK293 cells. Total RNA was extracted with TRIzol (Invitrogen product name), retrotranscribed with the AffinityScript RT (AffinityScript QPCR cDNA Synthesis Kit – Agilent), and the resulting cDNA was PCR-amplified using the previously described primers but without the restriction enzyme sites (forward: 5′-GGG AAC TGA AGA GAA AGT GAG G-3′) and (reverse: 5′-GGC TGA GTG AGT AGT TGT TTG C-3′). The amplified products were separated by electrophoresis on a 2% agarose gel and were subsequently analyzed by Sanger sequencing.

## Results

### Clinical features

**Figure 1:**
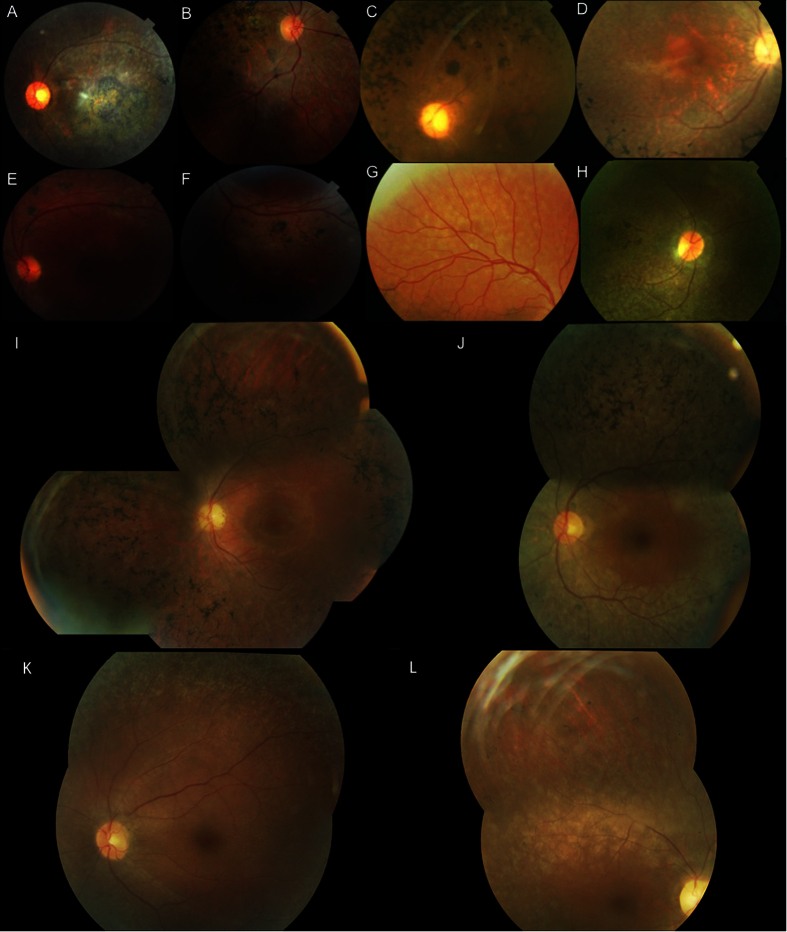
Clinical features of patients. Fundus photographs of (**A**) left eye of index patient (F8) with *PROM1* mutation; (**B**) right eye of IV-4 patient (F3) with *ABCA4* mutation; (**C**) left eye of index patient (F5) with *RPE65* mutation; (**D**) right eye of the index case (F2) with *CNGB1* mutation; (**E**,**F**) left eye of subject IV-1 in family F4 with *NR2E3* mutation; (**G**) right eye of subject VI-1 (F1) with *CNGB3* mutation; (**H**) right eye of IV-1patient (F6) with *RPE65* mutation; (**I**) left eye of index case IV-2 (F9) with *EYS* mutation, (**J**) left eye of subject III-9 (F10) with *PDE6B* mutation, (**K**) left eye of the proband V-1 (F7) with *RPE65* mutation, (**L**) right eye of subject V-1 (F11) with deletion in *FAM161A*.

As summarized in Table 2, patient phenotypes included RP and early-onset retinal degeneration (EORD) and STGD (Fig. 1). The clinical presentation of patients in families F2, F4, F5, F7, F8, F10 and F11 showed typical hallmarks of RP symptoms for which affected individuals initially experienced night blindness with progressive visual loss and hemeralopia in the majority of cases. Specifically, affected individual IV-5 (aged 40 years) of F2 family reported poor visual acuity. Funduscopy revealed typical RP changes with macular atrophy in F10 affected members suffering from photophobia; onset of symptoms was reported to occur during the first decade of life, with typical RP changes and normal macula. Fundus examination of patient III-10 (aged 36 years) of F10 revealed normal macular pattern with typical RP, with no nystagmus or hemeralopia. In F11, the index patient V-1 presented typical RP with atrophy of the retina and macular edema.

**Figure 2:**
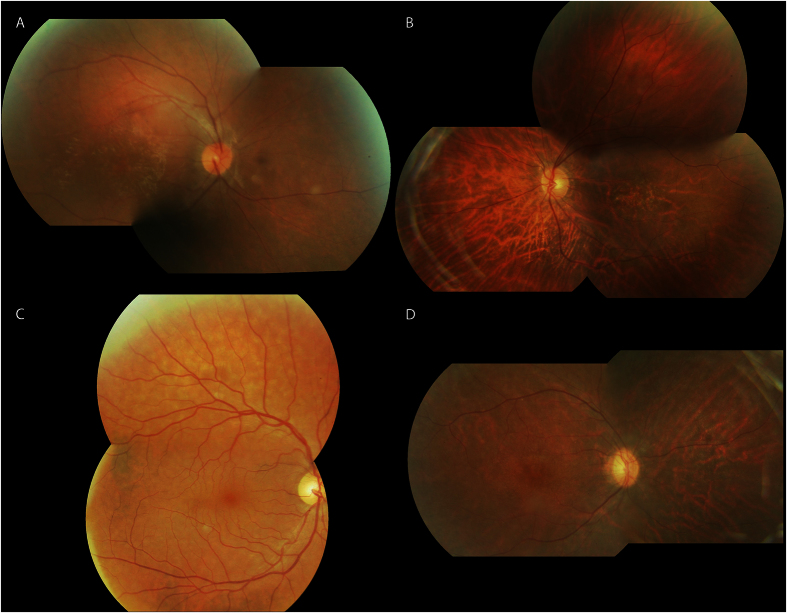
Spectrum of fundus findings among different families (F12, F13, F14, F15) with mutation in *CERKL*.

In addition, clinical data of F9, F12, F13, F14 and F15 (Fig. 2) showed typical hallmarks of RP symptoms for which affected individuals initially experienced night blindness with progressive visual loss and hemeralopia in the majority of cases. Fundoscopy revealed mild optic disc and retinal atrophy without pigmentation. Macula was normal in young patients and showed atrophic alteration in advanced stages.

Intermediate phenotypes between LCA and RP were observed in two families. In F1 and F6 the affected individuals had poor visual acuity at the ages of 10–12 years respectively, with a clinical picture suggesting EORD. Two retinal patterns were identified: pattern 1 presented mid-peripheral deep white dot deposits and virtually no clumped pigmentation (F1), whereas pattern 2 showed mid-peripheral pigmented clumps with no white deposits (F6). Family F3 presented a very advanced form of STGD. Night blindness was the first symptom, followed by intense photophobia. All had useful vision until the second decade of life. Fundus examination revealed atrophy of the macula, pallor of the optic disk and bone spicule-shaped pigment deposits in the periphery. SD-OCT show diffuse atrophy with Epiretinal membrane. Only family F3 presented a Stargardt phenotype. All cases presented non-syndromic phenotype (no associated extraocular abnormalities).

### Mutation analysis

Following the clinical diagnosis of retinal dystrophy, WES or IROme was performed to screen for all known genes and to look for novel candidate genes. Assuming identity-by-descent in these consanguineous families, we identified and examined autozygous regions when they spanned more than 5 Mb of genomic DNA. After all filtering steps, a few strong candidate genes remained in the autozygous regions of interest. Known RD genes were selected first for direct sequencing.

**Figure 3:**
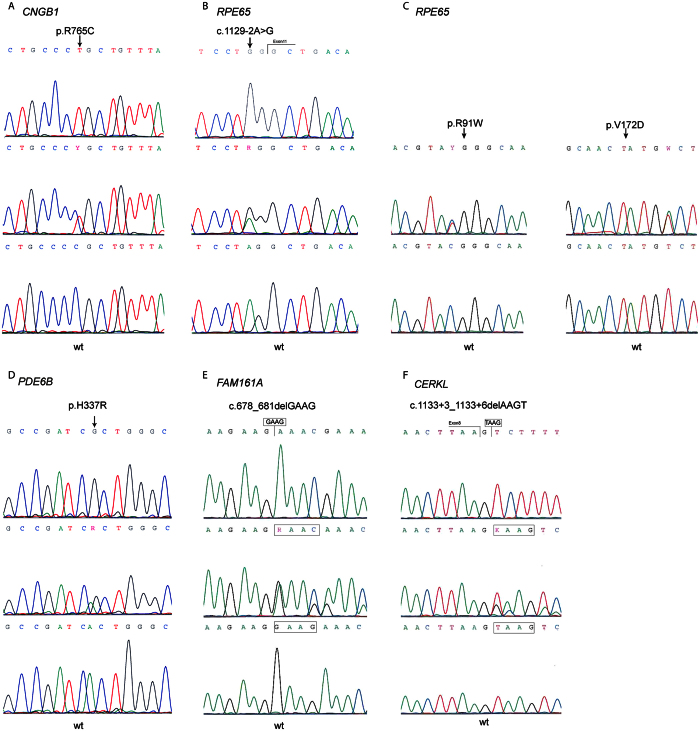
Chromatograms of the six novel mutations in *CNGB1*, *RPE65*, *PDE6B, FAM161A* and *CERKL*. For each mutation, wild-type (wt) sequence is depicted with the homozygous and heterozygous sequences. (**A**) c.2293C > T; p.R765C (*CNGB1*), (**B**) c.1129-2A > G (*RPE65*), (**C**) c.[325C > T];[569T > A] (*RPE65*), (**D**) c.1010A > G; p.H337R (*PDE6B*), (**E**) c.678_681delGAAG (*FAM161A*), and (**F**) c.1133 + 3_1133 + 6delAAGT(*CERKL*). Arrows indicate the position of the mutated nucleotide.

Mutation analysis in our patients revealed seven novel homozygous mutations. The first mutation found was a missense substitution, c.2293C > T, in exon 23 of *CNGB1* (NM_001297.4), resulting in p.R765C (Fig. 3A) detected in family F2. It is considered to be probably damaging with a score of 1.0 by polyphen-2 and R765 is conserved from mammals to Danio rerio (Zebrafish). The second variant was the substitution of A to G at nucleotide position 1010 in exon 7 of the *PDE6B* (NM_001145291.1) and resulted in the substitution of histidine (H) by arginine (R) at codon 337 (p.H337R) (Fig. 3D) in family F10. This variant is predicted to be benign with polyphen-2 (Table 3). This amino acid is also conserved from mammals to Danio rerio. These two mutations were amongst those included in the evaluation of IROme^8^.

**Figure 4:**
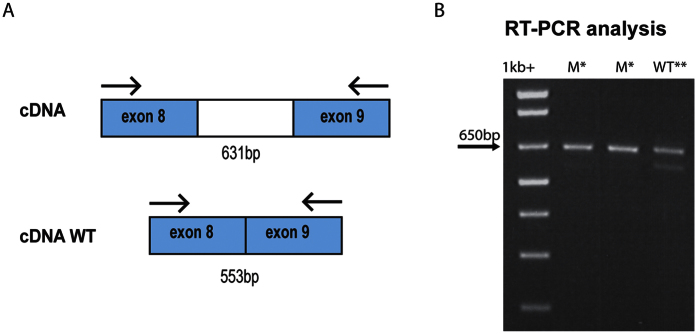
RT-PCR products with primers in exon 8 and 9. (**A**) Schematic representation of normally spliced mRNA, (**B**) Aberrantly spliced mRNA. In the gel electrophoresis we can observe absence of splicing in the mutated patient (column M). In the WT sequence (column WT), splicing was not complete. *Non-spliced fragment, **spliced fragment.

Two new mutations were found in *RPE65.* The first was in the splice site of *RPE65* at position c.1129-2 and changed the canonical splice site second nucleotide to G in family F6 (Fig. 3B). The second mutation was identified in family F7 and was a new compound heterozygous mutations c.[325C > T];[569T > A] resulting in p.(R91W);(V172D) (Fig. 3C). The V172D mutation was not described in ExAC Browser^9^, 1000 genomes^11^, and HGMD^10^ databases; this mutation is predicted to be possibly damaging with a score of 0.721. In family F11 we identified several genomic homozygous regions, one of which included *FAM161A*. Sequence analysis and validation by Sanger sequencing showed a new homozygous deletion of four nucleotides GAAG in exon 3 (c.678_681delGAAG) (Fig. 3E) resulting in a frameshift and a termination codon at position p.K227Nfs*17. To our knowledge, this mutation has not previously been reported in the literature or as variation in databases such as 1000 genomes^11^ and ExAC. The last mutation was a 4-bp deletion, c.1133 + 3_1133 + 6delAAGT, located in the donor splice site of intron 8 of *CERKL* in family F12 (Fig. 3F). This new deletion was present at a homozygous state in the two affected sons and was heterozygous in the parents. It was not present in the unaffected sister. Furthermore, this deletion affects the invariant acceptor site of exon 8, which has not previously been linked to this particular RP phenotype. Minigene analysis confirmed that this variant has a strong impact on the normal splicing pattern of *CERKL*. Transfection of HEK 293 cells with minigene constructs supporting this change and its wild-type counterpart revealed that c.1133 + 3_1133 + 6delAAGT did affect the canonical splicing of exon 8 by knocking down its natural 3′ splice site (Fig. 4).

As the fundus of affected F12 family members showed atrophy of the peripheral retina with no clumped pigmentation, we looked for this specific phenotype in families with simplex cases or where consanguinity was not evident. Fourteen additional Tunisian families were screened for this specific mutation. This allowed us to identify the homozygous c.1133 + 3_1133 + 6delAAGT deletion in three seemingly unrelated families (F13, F14, F15), but originating from the same geographical area. Genotype analysis of these three families showed a common homozygous region of 5.7 cM. This is indicative of an unknown consanguinity of these families (Table 4).

**Figure 5:**
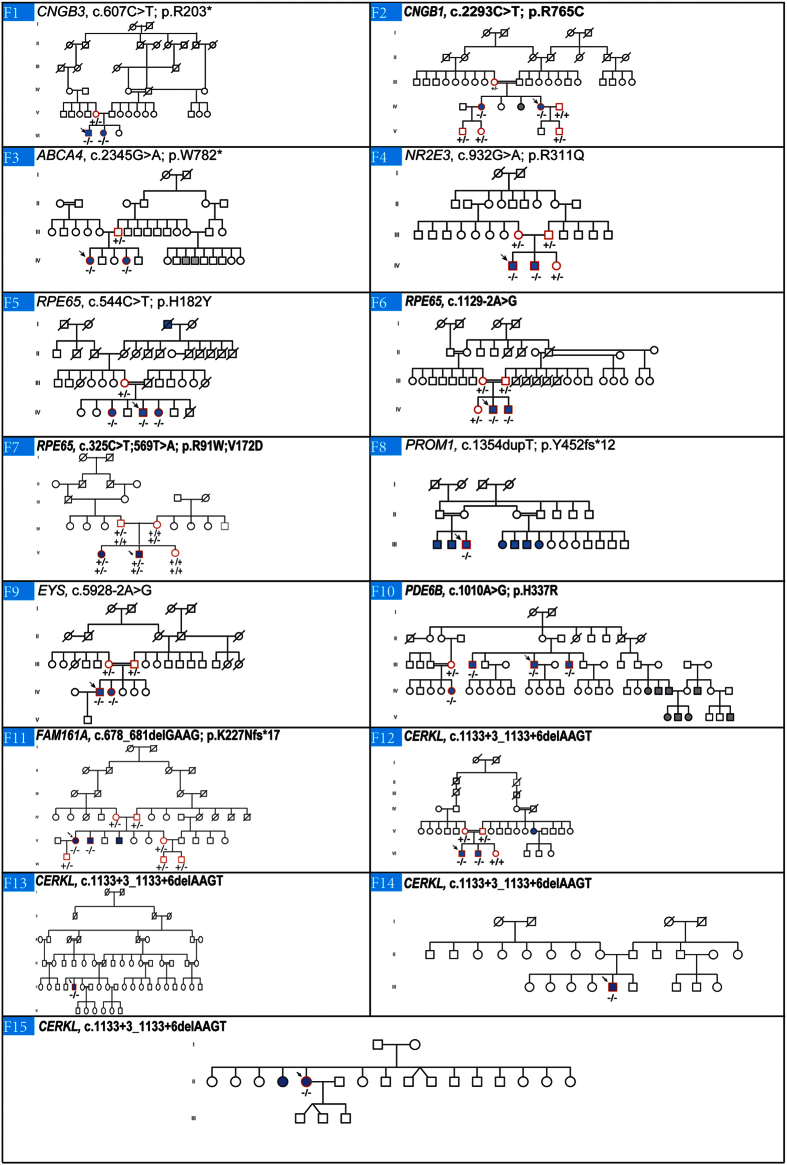
Overview of the pedigree structure of the families and segregation analysis of disease causing variants in the RD cohort. Arrows point to the probands. Affected individuals are indicated with filled symbols (blue), suspected (grey), whereas unaffected relatives are indicated by open symbols. +: wild type allele; −: mutation. All mutations are homozygous except for family F7.Genes with new mutations are in bold.

The mutations identified in the other families have already been reported in the literature. These changes were two nonsense substitutions: p.R203* in *CNGB3* (NM_019098.4) in family F1 and p.W782* in *ABCA4* (NM_000350.2) in family F3; two missense mutations: p.R311Q in *NR2E3* (NM_014249.3) in F4 and p.H182Y in *RPE65* (NM_000329.2) in F5, one frameshift mutation: c.1354dupT in *PROM1* (NM_001145848.1) in F8, and one splice site mutation: c.5928-2A > G in *EYS* (NM_001292009.1) in F9. A summary of these results is presented in Table 3. Cosegregation analysis in each family showed that all affected individuals were homozygotes and unaffected individuals were either heterozygous carriers or homozygous for the wild type allele (Fig. 5).

## Discussion

Molecular diagnosis of RD is a challenging task given the high genetic heterogeneity of this group of diseases. Aiming to identify the molecular origin of RD in our Tunisian population, we performed molecular analysis of index cases from Tunisian families and revealed a great genetic variety among this population. Ten different genes were involved in 15 families. Previous studies in the Tunisian population have shown a high genetic heterogeneity in RP patients^14^ as well as in patients with Usher syndrome^15^. This could be explained by the degree of consanguinity observed in various parts of the country. The genes identified play different roles in the retina. Most genes mutated in this sub-cohort are a part of three major processes in rod photoreceptors and RPE. The first three genes, *PDE6B*, *CNGB1* and *CNGB3*, are implicated in the phototransduction cascade: *PDE6B*, encoding the beta-subunit of rod cGMP-phosphodiesterase (cGMP-PDE), is a key enzyme of the retinal rod phototransduction cascade. *PDE6B* was reported by several groups as one of the causative gene associated with arRP, including previous studies of consanguineous Tunisian families^16^,^17^,^18^,^19^. Mutations in *CNGB1* encoding the beta-subunit of the rod cGMP-gated channel explain about 4% of autosomal recessive non-syndromic RP^4^. We discovered a new p.R765C homozygous missense substitution in exon 23 of *CNGB1* in F2. This mutation occurred at an evolutionarily conserved position from mammals to zebrafish and is predicted to damage the protein. So far, only eight point mutations were described with convincing evidence. Four of them were non-sense. Our result concurred with that of Bariel *et al.*, who found mutation in CNGB1 in a consanguineous French family affected with severe autosomal recessive RP (arRP)^20^. *CNGB3* encodes the β-subunit of the cone photoreceptor cGMP-gated channel, essential for phototransduction in all three classes of cones^21^. Mutations in this gene are a common cause of achromatopsia^14^. Missense mutations in *CNGB3* have previously been reported in two individuals with cone–rod dystrophy and in a single individual with a progressive cone dystrophy phenotype^22^. Although the p.R203* mutation was previously associated with achromatopsia^21^, in family F1 it induced a more severe phenotype that was classified as EORD. In the past, CNGB3 was mainly screened in patients with achromatopsia in which it represents a common cause. With current high-throughput sequencing, most approaches analyze a large set of genes. This non-biased approach will most likely widen the genotype-phenotype correlation of most diseases^23^,^24^,^25^.

We also observed four families with homozygous mutations in the retinoid cycle genes. In *ABCA4,* a member of the ABC transporter superfamily associated with STGD disease^26^, CRD, RP^27^,^28^ as well as age-related macular degeneration^29^, a p.W782* was identified in family 3 with STGD phenotype. In *RPE65,* a vitamin A trans-cis isomerase, a p.H182Y was observed in family 5. This mutation was first reported by Morimura *et al.* as a compound heterozygous mutations in a single family with two boys affected with LCA^30^. Interestingly, this amino acid has also been mutated into Asparagine and Arginine as reported in Hanein *et al.* and Jacobson *et al.*, respectively^31^,^32^. The p.H182N and p.H182R were associated with LCA^31^,^32^. Unfortunately, there are not enough families with mutations at H182 to establish a strong genotype-phenotype correlation for this position. Mutation analysis in the region of homozygosity in family F6 revealed a new mutation c.1129-2A > G that affects the consensus splice site of exon 11 of *RPE65* and segregates with EORD. A compound heterozygous mutation p.(R91W);(V172D) was discovered in one family (F7). To date, a number of different *RPE65* mutations have been reported in patients with retinal dystrophies classified as LCA, autosomal recessive EORD or arRP^30^,^33^,^34^. However, in our cohort we found three different *RPE65* mutations in three different families that correlated with typical RP phenotype despite the early age of onset. Morimura *et al.*, reported a family in which a child with LCA phenotype is the offspring of two parents with RP^23^. A recent Chinese study, including 88 candidate genes in 179 families with RD, confirmed that the average age at onset was 5 years in patients carrying mutation in *RPE65* gene^24^. In patients from family F4 harboring the p.R311Q, the most prevalent mutation in the nuclear receptor subfamily 2, group E, member 3 (NR2E3), we observed typical RP with macular atrophy, as already reported^35^. Nevertheless, Gerber *et al.* have shown that the p.R311Q mutation could cause late-onset arRP in Crypto-Jewish population^36^. *NR2E3* recessive mutations are usually associated with enhanced S-cone syndrome, an autosomal recessive retinopathy in which patients have increased sensitivity to the perception of blue light while the dominant p.G56R mutation is associated with severe RP^21^,^22^. A homozygous splice alteration in EYS (c.5928-2A > G) was reported in family F8^23^. EYS is likely to play a role in the modeling of retinal architecture^37^. In previous studies, various mutations including point mutations, splice-site mutations and gross rearrangements were identified in patients with arRP^23^,^38^,^39^,^40^ and cone rod dystrophies^41^. The phenotype observed in family F8 resembles the RP phenotype reported by Barragan *et al.*^38^ in the Spanish population. The advanced age of onset of the disease could be correlated with our molecular result, as Huang *et al.* found a relationship between the advanced average age at onset and *EYS* mutation^24^. The mutation in *EYS* appears to be a frequent cause of arRP (15.9%) in the Spanish population^28^. Unfortunately, the prevalence of *EYS* mutations remains to be established in Tunisian population. Our preliminary analysis of the mutation load in the Tunisian population does not allow us to draw a similar conclusion. The fifteen families described here represent a selection that underwent whole-exome sequencing and thus do not represent a correct estimation of the yield of this approach.

In addition, a previously described variant (c.1354dupT) in *PROM1*, which encodes a pentaspan transmembrane glycoprotein that maintains the structural integrity of retina, was observed in patient III:1 from family F7 affected with RP^42^. Mutations in *PROM1* are usually associated with RP or CRD, which is the case in this family^43^.

We also report the identification of a new small deletion in exon 3 of *FAM161A*, a ciliary gene previously associated with RP^44^. This mutation causes a shift in the reading frame resulting in a premature termination codon at position p.K227Nfs*17, presumably inhibiting protein production due to the action of the nonsense mediated mRNA decay. Most currently known mutations^44^,^45^ cluster in exon3 of *FAM161A*, which is by far the largest coding exon of this gene. FAM161A is member of the growing list of ciliary proteins implicated in human diseases. Phenotypes of ciliopathies are quite diverse and may involve impairment of multiple organs or functions such as kidney, brain, bones, obesity and vision^46^. However, in the patients investigated in this study we did not observe any other obvious features or anamnestic history of extraocular symptoms typically related to ciliopathies. The phenotype was typical of RP.

Finally, it is of note that the analysis of four families (F13, F14, F15 and F16) originating from the same Tunisian region revealed a new homozygous deletion c.1133 + 3_1133 + 6delAAGT in the Ceramide kinase Like (*CERKL*) gene in association with RP without pigmentation. Mutations in *CERKL* have been described in RP (RP26) together with significant macular involvement during the early stages of the disease^47^ and cone-rod dystrophy which progresses to an RP-like phenotype in advanced stage^48^. However, the exact function of *CERKL* remains unknown. Genotype analysis of these four families showed a common disease-associated haplotype and supports the hypothesis of a common ancestor in this area.

Despite intensive research and studies, it is still very difficult to screen for specific genes based on clinical observation and severity of disease. Molecular diagnosis of RD is a challenging task given the important genetic heterogeneity of this group of diseases.

## Additional Information

**How to cite this article**: Habibi, I. *et al.* Identifying mutations in Tunisian families with retinal dystrophy. *Sci. Rep.* **6**, 37455; doi: 10.1038/srep37455 (2016).

**Publisher's note:** Springer Nature remains neutral with regard to jurisdictional claims in published maps and institutional affiliations.

## Figures and Tables

**Table 1:** Sequences of primers and PCR condition.

Genes	Exon	Forward	Reverse	Hybridation temp. (°C)
*CNGB3*	5	GTGAGAACATGCGGTGTTTG	CAAAGATGGGCAAATGATCC	58
*CNGB1*	23	AGAGACTCCGCCTCTCACTC	GGGGCAGACACGAAGATG	58
*ABCA4*	15	AGCACATGGAGTGTGCGTAG	TGCCCCTGTACATTTTAGCC	58
*NR2E3*	6	TCTGAGCCTCTGGCTGATGTCA	AGAAGGGAGTCCAGCCTCAC	60
*RPE65*	4	GACTTGATGAGGACACATAG	CAGTCCAGTAATTTTCAAGC	60
*RPE65*	6	TTCAAGGGGTAGTGATGACC	GCACAAAATGCTATTCTGAC	60
*RPE65*	11	CTTAGGAGCCAAGACTTAAG	GAGGAAACTCAAATGCTACG	60
*PROM1*	12	ctccagccttagtccagcag	gtcccatcacagcaggatct	58
*EYS*	29	AATCTGCTTCTGGCTTTGTTT	GCCCCACTAGCCAGAAAATA	58
*PDE6B*	7	CCTGCACACAGACATCCAGT	TGGCAGAGACAAGGAGAAGC	58
*FAM161A*	3	TGGTCACATACAACTGAAAGTATAACA	GCTTCTGTTCCTCCCTTGCT	60
*CERKL*	8	GGGAACTGAAGAGAAAGTGAGG	GGCTGAGTGAGTAGTTGTTTGC	58
*LRP2*	69	TTTTTGCTCCCCATCTTCTG	AATTCAGCAGGTGGGAGTTG	58
*PPIG*	14	CATTTCCCCAATTCTTTTTCTT	GATTGTGCGCGTCTATCCTT	58
*CWC22*	10	GCTCCTGGAAAGACCAACAG	GCCTCTTTCCAACTAACATGC	58
*ITGA4*	24	AAAACCAGGTTGCTTTTTGC	ACCATGCTTGTTCCTTCCAC	58
*PDE1A*	7	ACAGGGTGCACTGAAAGTCC	TTCTGGGCAGAGACAGATTG	58
*NCKAP1*	11	AGCAACATAAGAAAGGCAGGA	GAAGAATGCAGGGAGGTACG	58
*ZNF804A*	4	CCACTGTTGCTGAAGATCCA	TTTGATCTGCTAATGGGACTGA	58
*FSIP2*	12	ATCCCTGATTGTGGCAATTC	AAGCAGACTGTTCAAGGGCTA	58
*COL3A1*	30	TAGTTCCCACCCAGCTGTTC	CAGCAGCACCCTGAAAATAA	58
*ANKAR*	16	TGTCAATATAGCATACCCCTTTG	TTTTTGGCTTATTTCTCCAAGA	58

**Table 2:**
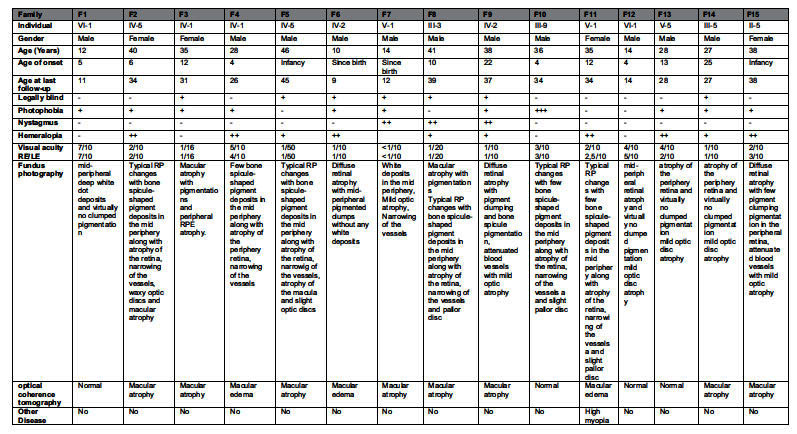
Clinical features of the patients examined.

+ and − symbols indicate presence/absence, as well as degree of a given feature (+ mild, ++ moderate, +++ severe). F: family; RE: right eye; LE: Left eye.

**Table 3:** Homozygous regions and mutations identified in this study.

Family ID	Disease	Genotyping Method	Size of homozygous region, in Mb	Chromosome	Gene	DNA mutation	Predicted protein variant	Coverage	Reference sequence	Previously reported	SIFT	Polyphen
**F1**	Eord	WES	30.4	8q21.3	*CNGB3*	c.[607C > T];[607C > T]	p.(R203*);(R203*)	26	NM_019098.4	[21]	—	—
**F2**	RP	IROme	—	16q21	*CNGB1*	c.[2293C > T];[2293C > T]	p.(R765C); (R765C)	—	NM_001297.4	**This study** and [8]	0	1,00
**F3**	Stargardt	WES	5.8	1p22.1	*ABCA4*	c.[2345G > A];[2345G > A]	p.(W782*); (W782*)	5	NM_000350.2	[28]	—	—
**F4**	RP	WES	12.2	15q23	*NR2E3*	c.[932G > A];[932G > A]	p.(R311Q); (R311Q)	24	NM_014249.3	[35]	0.10	0.627
**F5**	RP	WES	54.1	1p31.3-p31.2	*RPE65*	c.[544C > T];[544C > T]	p.(H182Y); (H182Y)	36	NM_000329.2	[30]	0	0.872
**F6**	Eord	WES	18.2	1p31.3-p31.2	*RPE65*	c.[1129-2A > G];[1129-2A > G]	—	30	NM_000329.2	**This study**	—	—
**F7**	RP	IROme	—	1p31.3-p31.2	*RPE65*	c.[325C > T];[569T > A]	p.(R91W); (V172D)	—	NM_000329.2	**This study**	0.01/0.01	0.997/0.721
**F8**	RP	WES	9	4p15.32	*PROM1*	c.[1354dupT];[1354dupT]	p.(Y452fs*12); (Y452fs*12)	12	NM_001145848.1	[42]	—	—
**F9**	LCA	WES	58	6q12	*EYS*	c.[5928-2A > G];[5928-2A > G]	—	24	NM_001292009.1	[23]	—	—
**F10**	RP	IROme	—	4p16.3	*PDE6B*	c.[1010A > G];[1010A > G]	p.(H337R); (H337R)	—	NM_001145291.1	**This study** and [8]	1.00	0.046
**F11**	RP	WES	13	2p15	*FAM161A*	c.[678_681delGAAG]; [678_681delGAAG]	p.(K227Nfs*17); (K227Nfs*17)	94	NM_001201543	**This study**	—	—
**F12**	RP	WES	8	2q31.3	*CERKL*	c.[1133 + 3-1133 + 6delAAGT]; [1133 + 3-1133 + 6delAAGT]	—	21	NM_201548.4	**This study**	—	—
**F13**	RP	—	—	2q31.3	*CERKL*	c.[1133 + 3-1133 + 6delAAGT]; [1133 + 3-1133 + 6delAAGT]	—	—	NM_201548.4	**This study**	—	—
**F14**	RP	—	—	2q31.3	*CERKL*	c.[1133 + 3-1133 + 6delAAGT]; [1133 + 3-1133 + 6delAAGT]	—	—	NM_201548.4	**This study**	—	—
**F15**	RP	—	—	2q31.3	*CERKL*	c.[1133 + 3-1133 + 6delAAGT]; [1133 + 3-1133 + 6delAAGT]	—	—	NM_201548.4	**This study**	—	—

**Table 4:** Common homozygous genomic region.

rs.	Distance position	F12 (VI-1)	F13 (V-5)	F14 (III-5)	F15 (II-5)
rs4667591	−12.4 M	A/C	C/C	A/C	A/C
rs1050354	−11.9 M	T/A	A/A	T/A	T/A
rs4894140	−1.5 M	C/C	C/C	C/C	C/C
rs1143676	−0.18 M	A/A	A/A	A/A	A/A
*CERKL*	**0**	—	—	—	—
rs3754929	+0.67 M	T/T	T/T	T/T	T/T
rs1400130	+1.43 M	G/G	G/G	G/G	G/G
rs12476147	+3.3 M	T/T	T/T	T/T	T/T
rs17228441	+4.2 M	C/C	C/C	C/C	C/C
rs1800255	+7.4 M	G/A	G/A	G/A	G/A
rs1225090	+8.1 M	G/T	T/T	G/T	G/T
