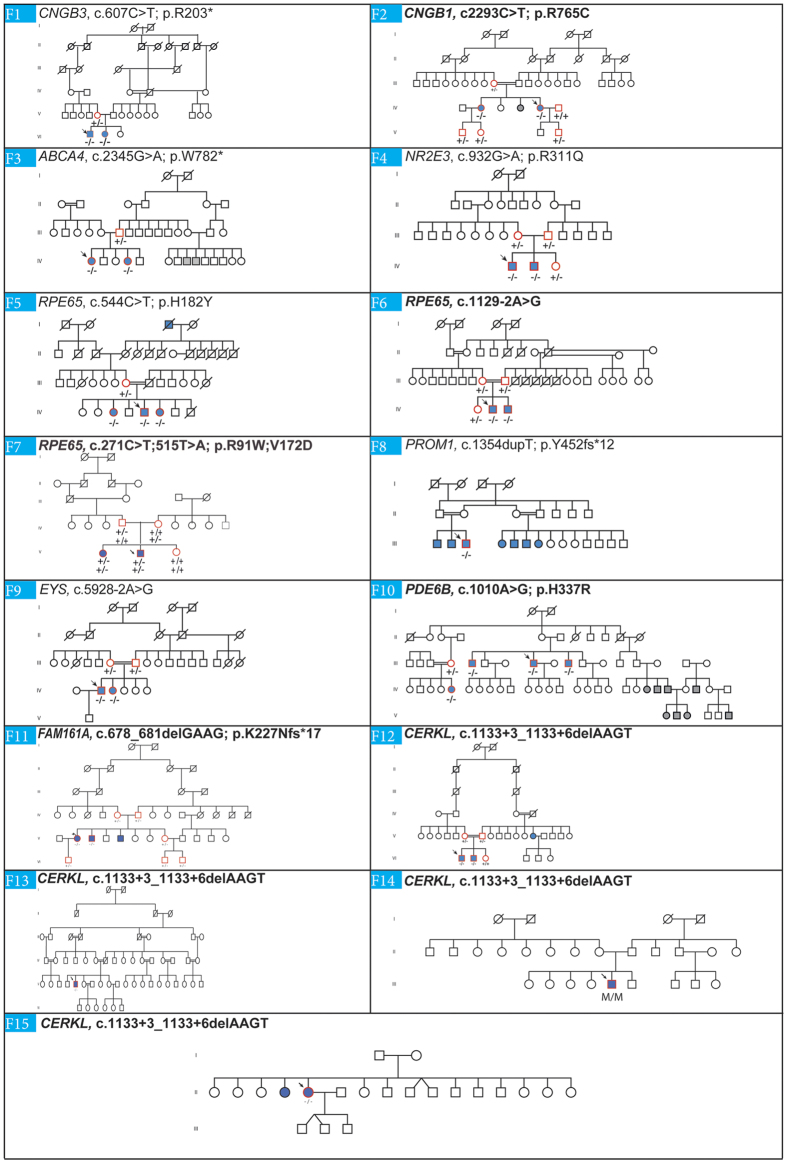# Corrigendum: Identifying mutations in Tunisian families with retinal dystrophy

**DOI:** 10.1038/srep46776

**Published:** 2017-05-04

**Authors:** Imen Habibi, Ahmed Chebil, Yosra Falfoul, Nathalie Allaman-Pillet, Fedra Kort, Daniel F. Schorderet, Leila El Matri

Scientific Reports 6: Article number: 3745510.1038/srep37455; published online: 11 22 2016; updated: 05 04 2017

This Article contains errors. The position of the mutation p.(R91W); (V172D) was incorrectly calculated, taking as a starting point the beginning of cDNA rather than the start codon. The correct position of the mutation is c.[271C > T]; [515T > A]. As a result of this the following changes in the Article are made:

The legend of Figure 3 is incorrect,

“(C) c.[325C > T]; [569T > A] (RPE65)”

should read:

“(C) c.[271C > T]; [515T > A] (RPE65)”.

In Table 3 for the family ID F7, the DNA mutation should read ‘c.[271C > T]; [515T > A]’.

In Figure 5 for family F7 ‘RPE65, c.325C > T; 569T > A; p.R91W; V172D’ should read ‘RPE65, c.271C > T; 515T > A; p.R91W; V172D’. The correct Figure 5 appears below as Figure 1.

In the Results section under the subheading ‘Mutation analysis’,

“The second mutation was identified in family F7 and was a new compound heterozygous mutations c.[325C > T]; [569T > A] resulting in p.(R91W); (V172D) (Fig. 3C)”.

should read:

“The second mutation was identified in family F7 and was a new compound heterozygous mutations c.[271C > T]; [515T > A] resulting in p.(R91W); (V172D) (Fig. 3C)”.

## Figures and Tables

**Figure 1:**